# Agricultural pesticides and ectoparasites: potential combined effects on the physiology of a declining aerial insectivore

**DOI:** 10.1093/conphys/coab025

**Published:** 2021-04-28

**Authors:** Audrey Sigouin, Marc Bélisle, Dany Garant, Fanie Pelletier

**Affiliations:** Département de Biologie, Université de Sherbrooke, Sherbrooke, Quebec J1K 2R1, Canada

**Keywords:** Ecotoxicology, ectoparasites, farmlands, pesticides, *Protocalliphora* sp, tree swallow

## Abstract

Agricultural pesticides usage has been increasing globally. These compounds have been developed to disrupt pest species physiology, but because their specificity is limited, they can also have adverse effects on non-target organisms. Recent studies have shown that the damaging toxicological effects of pesticides can be amplified in stressful environments. However, few studies have documented these effects in natural settings where organisms are simultaneously exposed to pesticides and to other environmental stressors such as parasites. In this study, we assessed both pesticide and ectoparasite effects on the physiology of a free-ranging bird. We measured physiological markers including haematocrit, bacteria-killing ability (BKA) and leucocyte counts, as well as exposure to haematophagous *Protocalliphora* larvae, in tree swallow nestlings (*Tachycineta bicolor*), a declining aerial insectivore, in southern Québec, Canada, for over 3 years. We found that combined exposure to pesticides and *Protocalliphora* larvae was negatively related to haematocrit, suggesting possible synergistic effects. However, we found no such relationships with BKA and leucocyte counts, highlighting the complexity of physiological responses to multiple stressors in natural settings. Populations of several aerial insectivores are declining, and although sublethal pesticide effects on physiology are suspected, our results suggest that exposure to other factors, such as parasitism, should also be considered to fully assess these effects, especially because pesticides are increasingly present in the environment.

## Teaser

In this study, we investigated whether exposure to pesticides and ectoparasites led to combined effects on tree swallow (*Tachycineta bicolor*) nestling physiology in an agricultural landscape. Our results show that haematocrit, but not bacteria-killing ability or leucocyte counts, was negatively affected by the combined exposure to these two stressors.

## Introduction

Human activities are increasingly affecting natural environments. An important human-driven environmental change is the intensification of agricultural practices (Green *et al.*, 2005). Over the past several decades, conversion of grasslands into row crop monocultures, simpler crop rotation and increased use of machinery and agrochemicals, has led to the simplification and homogenization of agricultural landscapes in many countries (Benton *et al.*, 2003; Stanton *et al.*, 2018). As a result, a wide range of species found in agricultural areas, including insects (Hallmann *et al.*, 2017; Wagner, 2019), amphibians (Stuart *et al.*, 2004) and birds (Murphy, 2003; Hallmann *et al.*, 2014; Rosenberg *et al.*, 2019), is declining worldwide. The drivers of these declines are often complex, but increased pesticide use has been hypothesized to play a crucial role. In Europe and North America, several studies suggest that sharp declines in farmland bird populations could be attributed to decreases in insect prey abundance (Campbell *et al.*, 1997; Benton *et al.*, 2002; Hart *et al.*, 2006; Stanton *et al.*, 2018; Møller, 2019) and to pesticide’s direct toxic effects (Donald *et al.*, 2001, 2006; Mineau and Whiteside, 2013; Stanton *et al.*, 2018; Spiller and Dettmers, 2019). Pesticides developed for agricultural purposes are increasingly toxic and tend to accumulate in the environment (Dibartolomeis *et al.*, 2019; Malaj *et al.*, 2020), leading to an urgent need to further assess their effects on wildlife (Gibbons *et al.*, 2014; Pisa *et al.*, 2014).

Pesticides are designed to be toxic for pests. Their toxicity, however, generally stems from the disruption of basic cellular and physiological processes shared by many taxa (Gibbons *et al.*, 2014; Zaller and Brühl, 2019). Therefore, pesticide specificity is limited and these substances can also affect non-target organisms. Acute exposure to pesticides leading to mortality in non-target wildlife has been reported (Kwon *et al.*, 2004; Rogers *et al.*, 2019), though current interest lies in the sublethal effects of chronic pesticide exposure (Bright *et al.*, 2008; Köhler and Triebskorn, 2013). For instance, carbamate and organophosphate insecticides are designed to inactivate the action of acetylcholinesterase, an enzyme that quickly catabolizes acetylcholine from synapses to prevent permanent firing of nervous impulses. This leads to targeted insect mortality but can also affect any exposed animal (Burgess *et al.*, 1999; Bishop *et al.*, 2000; Mineau and Tucker, 2002). Similarly, neonicotinoid insecticides bind to nicotinic acetylcholine receptors and overstimulate the nervous system, affecting physiological and behavioural processes in birds that impact their immunity, reproduction and migration (Lopez-Antia *et al.*, 2015b; Eng *et al.*, 2017, 2019). Atrazine, a globally used herbicide (Solomon *et al.*, 1996), is an endocrine disruptor (reviewed in Mnif *et al.*, 2011) and has negative effects on amphibian and fish immunity, including leucocyte number reduction and lymphoid organ atrophy (reviewed in Rohr and McCoy, 2010).

A major limitation to our understanding toxic pesticide effects on wildlife is that most studies are conducted under controlled laboratory conditions that might not be representative of natural environmental conditions. For example, laboratory assays usually consider the toxicity of high concentrations of active ingredients over short time periods (generally 30 days and up to 90 days maximum), whereas long-term exposure to low concentrations is more typical of natural settings (Mineau, 2005; Cox and Surgan, 2006). Laboratory studies also generally focus on exposure to a single active ingredient and rarely consider what happens with simultaneous exposure to multiple compounds (i.e. active agents or additives) that can act antagonistically or synergistically (Cedergreen, 2014; Hua and Relyea, 2014; Lebrun *et al.*, 2020). For example, atrazine amplifies the toxicity of other pesticides such as organophosphates (Belden and Lydy, 2000). Thus, to gain a better understanding of pesticide effects on wildlife, it is essential to study such toxicological effects in natural systems (Mineau and Palmer, 2013; Brühl and Zaller, 2019).

Another problem with simplified assays of pesticide effects performed under controlled conditions is that they rarely consider complex interactions with other environmental stressors (Blus and Henny, 1997; Holmstrup *et al.*, 2010). In the wild, organisms that are exposed to multiple stressors (natural and/or anthropogenic) could suffer stronger negative effects than if exposed to a single stressor (Holmstrup *et al.*, 2010; Marcogliese and Pietrock, 2011). Pesticides can have detrimental effects on their own, yet a growing body of literature highlights the importance of considering multiple environmental stressors, such as pathogens and parasites, to correctly assess contaminant effects (Marcogliese and Pietrock, 2011; Sures *et al.*, 2017). For instance, combined exposure to pesticides and parasites could impair the immune reactions of hosts towards pathogens (Coors *et al.*, 2008; Marcogliese *et al.*, 2009; Booton *et al.*, 2018), enhance parasite intensity (Gentes *et al.*, 2007) or lead to synergistic negative effects on physiological processes (Sures, 2008; Marcogliese *et al.*, 2010; Marteinson *et al.*, 2017). To date, most studies on such combined effects have been conducted on aquatic (*Salvelinus alpinus*, Blanar *et al.*, 2005; *Perca flavescens*, Marcogliese *et al.*, 2010) or semi-aquatic (*Lithobates catesbeianus*, Marcogliese *et al.*, 2009; King *et al.*, 2010) species and very few have been conducted on terrestrial vertebrates, such as birds (but see Eeva *et al.*, 1994; Gentes *et al.*, 2007; Marteinson *et al.*, 2017). Thus, a better understanding of multiple environmental stressor effects on physiology and immune function is needed and could prove especially important in identifying the underlying causes of population declines, such as in amphibians (Hayes *et al.*, 2010; Blaustein *et al.*, 2011) and in birds (Rosenberg *et al.*, 2019; Spiller and Dettmers, 2019).

The lack of ecotoxicological field studies considering both pesticides and parasites effects is due to the need of individual level data on exposures and responses to multiple stressors through time and/or across multiple sites. Our goal was to investigate both agricultural pesticide and ectoparasite effects on tree swallow (*Tachycineta bicolor*) nestling physiology for over 3 years. Similar to other aerial insectivore populations, tree swallows are declining in northeastern North America (Nebel *et al.*, 2010; Shutler *et al.*, 2012; Smith *et al.*, 2015; Michel *et al.*, 2016). Although agricultural intensification is hypothesized to play a role in these declines, specific drivers and mechanisms are still poorly understood. Tree swallow nestlings, like most altricial birds, are often infested by parasites, including haematophagous *Protocalliphora* fly larvae (Bennett and Whitworth, 1991). Although several studies reported negative effects of *Protocalliphora* on nestling physiology (Sabrosky *et al.*, 1989; Whitworth and Bennett, 1992; Simon *et al.*, 2004; Dawson *et al.*, 2005; Thomas *et al.*, 2007), others have found little or no effects (Howe, 1992; Hannam, 2006). Furthermore, studies addressing pesticide effects on free-ranging passerine bird immunity remains limited. Bishop *et al.* (1998) found that tree swallows nesting in orchards in Ontario, Canada, had lower haematocrit but an enhanced immune response to pesticide exposure, indicating a possible autoimmune reaction (see also Mayne *et al.*, 2004, 2005).

To assess both pesticide and ectoparasite effects on tree swallow nestling physiology, we used three markers: haematocrit (% volume of red blood cells), bacteria-killing ability (BKA) and differential leucocyte counts (including granulocytes, lymphocytes and monocytes). We chose these markers because they are suitable indicators of physiological performance and immunocompetence in birds (Lobato *et al.*, 2005; Matson *et al.*, 2006; Norte *et al.*, 2009; Boughton *et al.*, 2011). These markers represent several different components of the immune system because wildlife generates different immune responses to different pathogen types (Sheldon and Verhulst, 1996; Lochmiller and Deerenberg, 2000). Previously, we found that landscape habitat composition linked to agricultural intensification affected immune response in tree swallows (Pigeon *et al.*, 2013a,b; Schmitt *et al.*, 2017), suggesting a possible impact of pesticides on these markers. However, these studies did not directly investigate these specific physiological markers or potential combined pesticide and parasite effects on them.

We hypothesized that exposure to both pesticides and haematophagous ectoparasites could have additive or synergistic effects on tree swallow nestling’s physiological markers. Physiological homeostasis is costly to maintain, so nestlings would not be able to cope efficiently with both the toxic effects of pesticides and the consequences of parasitism. We therefore predicted that haematocrit, BKA and leucocyte counts in nestlings would decline in response to the combined exposure of pesticides and parasitism. Decreased physiological performance could ultimately affect nestling growth and survival (Christe *et al.*, 1998; Møller and Saino, 2004; Lobato *et al.*, 2005; Bowers *et al.*, 2014).

## Materials and methods

### Study area and population

**Figure 1:**
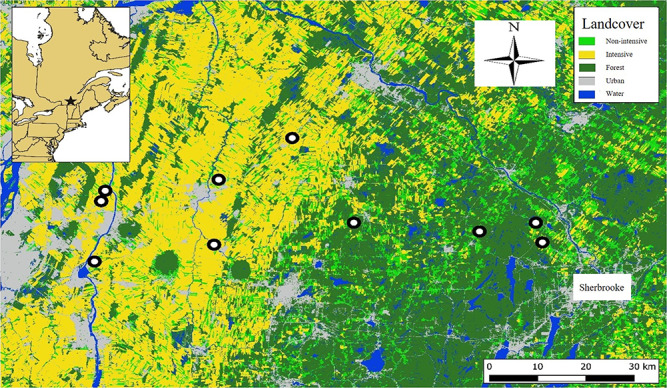
A map of our study system in southern Québec, Canada. The top left box illustrates the location (star) of our study system in Québec. The 10 farms considered in this study are represented by the white dots. ‘Non-intensive’ and ‘Intensive’ refer to agricultural practices and crop types.

Tree swallows are migratory aerial insectivorous passerines that breed over most of North America (Winkler *et al.*, 2011). Here, we capitalize on a population of tree swallows in southern Québec, Canada, that have been monitored annually since 2004. Tree swallow breeding activity was assessed over a network of 400 nest boxes distributed equally among 40 farms across an area of 10 200 km^2^ (Ghilain and Bélisle, 2008). In 2013–2015, we monitored physiological markers for a subsample of 100 nest boxes on 10 farms: 4 were located in non-intensively cultivated environments and 6 in intensively cultivated environments (Fig. 1). These farms are distributed along an east–west agricultural intensification gradient, with non-intensively cultivated crops (i.e. cattle foraging crops such as hay, alfalfa and clover, as well as pastures) dominating in the east and intensively cultivated row crops (i.e. monocultures of corn, soybeans and other cereals) in the west. Farms with intensively cultivated row crops are poorer breeding habitats for tree swallows and this might negatively affect their fitness (Ghilain and Bélisle, 2008; Lessard *et al.*, 2014) because increased pesticide use (Giroux, 2019; Montiel-León *et al.*, 2019; Poisson, 2019) can lead to lower prey availability (Rioux Paquette *et al.*, 2013; Bellavance *et al.*, 2018). Nest boxes were monitored every 2 days from adult arrival (end of April) to end of fledging (end of July). In a given clutch, eggs are incubated for ~12 days, all nestlings usually hatch within a 24-hour period (Quinney *et al.*, 1986) and nestlings fledge 20–22 days after hatching (Winkler *et al.*, 2011). Birds were captured, handled and banded in compliance with the Canadian Council on Animal Care, under the approval of the Université de Sherbrooke’s Animal Ethics Committee (protocols MB2018-01 and FP2018-01 of the Université de Sherbrooke).

We assessed nestling exposure to pesticides via nestling diet by collecting boluses of insects brought by parents to the nest (Bellavance *et al.*, 2018). Boluses were collected from nestlings aged 6, 8 and 10 days during two consecutive 30-min sessions on each sampling occasion. Collection occurred at the end of each 30-min session and stopped when we obtained a total of 5 boluses for a specific nest box or a total of 10 boluses for a farm (whichever came first) to reduce the impact of this manipulation on nestlings. As a result, 34% of the broods sampled for boluses were ligatured for just one 1-hour session, 48% for two 1-hour sessions and 18% for three 1-hour sessions. A previous study by Bellavance *et al.* (2018) found no differences in fledging probability between nestlings manipulated under such a sampling regime and those that were not. Each bolus was kept in individual, sterile Falcon® tubes and kept on ice in the field for <12 hours, transferred to −20°C for no longer than 1 week, and then stored at −80°C until laboratory analysis.

**Figure 2:**
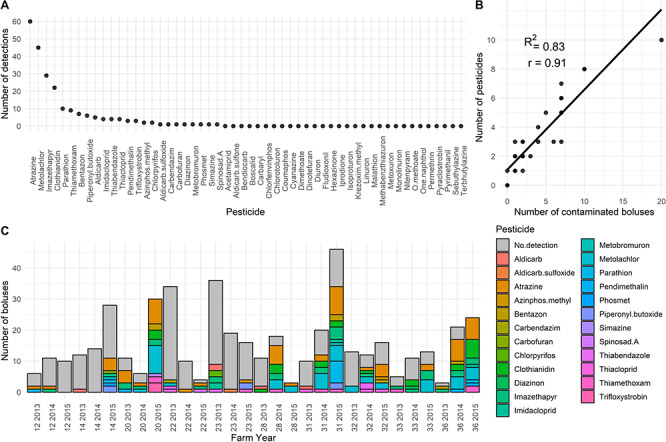
Descriptive statistics on pesticides analysed in insect boluses fed to tree swallow nestlings in southern Québec, Canada, 2013–2015. (A) Number of detections over the entire study area of the 54 pesticides considered in our chemical analysis (Haroune *et al.*, 2015). (B) Number of boluses contaminated by at least one pesticide as a function of number of pesticides detected at the farm level. (C) Count of boluses in which specific pesticides were or were not detected for all farm-year combinations (the first two digits refer to the farm number and the last four indicate the year of sampling). Pesticides never detected are not listed in the legend.

For each insect bolus, we assessed the presence and concentration of 51 pesticides and 3 of their derivatives (hereafter referred to as pesticides for simplicity) using a microwave-assisted solvent extraction and a salt-out effect method (Haroune *et al.*, 2015). We used ultra-high-pressure liquid chromatography–tandem mass spectrometry (see Haroune *et al*., 2015, for details) for compound identification. We considered a list of pesticides that had diverse chemical classes (9 fungicides, 18 herbicides and 24 insecticides) and modes of toxicity (10 organophosphates, 7 carbamates (+3 derivatives) and 7 neonicotinoids). A detailed list of compounds and their respective detection and quantification limits can be found in Table S1. All pesticides we screened for are used or have been used in our study area (Giroux, 2019; Montiel-León *et al.*, 2019) and have adverse effects on animals (Blakley *et al.*, 1999; Galloway and Handy, 2003; Köhler and Triebskorn, 2013; Gibbons *et al.*, 2014). Several compounds were not detected (Fig. 2), so we pooled the number of detections at the farm-year level for subsequent statistical analysis. This was done assuming that nestlings raised on the same farm would be exposed to pesticides with similar intensity.

### Parasite counts

*Protocalliphora* is a genus of calliphorid flies (Diptera: Calliphoridae) with a parasitic haematophagous larval stage that is very common in altricial bird nests (Sabrosky *et al.*, 1989). Adult female flies lay their eggs in the bird nest, and larvae emerge to feed on nestlings before pupating. After 14–21 days, adults emerge from pupae (Bennett and Whitworth, 1991) leaving empty shells (puparia) in a host’s nest. Each year, after breeding season was complete, entire tree swallow nests were collected and stored in plastic bags at 4°C. One day prior to nest sorting, nests were stored at −80°C to kill any parasites. Nests were weighed (± 0.01 g) (P-2002, Denver instrument, Bohemia, NY, USA), and then under a ventilated hood, we sorted nest material to collect *Protocalliphora* pupae and empty puparia. For each nest, we counted pupae and puparia and preserved them in 75% ethanol. We know that *Protocalliphora sialia* is the dominant species in our nest box system (96.5% and 87.6% of infested nests in 2008 and 2009, respectively) but that *Protocalliphora metallica* and *Protocalliphora bennetti* also parasitized our tree swallow nests (Daoust *et al.*, 2012). Because we did not identify pupae and puparia to species level for this study and because no data are available on relative effect of each species, all specimens were grouped under *Protocalliphora* spp., hereafter referred to as *Protocalliphora*.

### Blood sampling and haematocrit measures

Haematocrit has been linked to individual physiological performance in several studies (Thomas *et al.*, 2007; Norte *et al.*, 2010) and is key when oxygen uptake is important during both nestling growth and just after fledging (Puerta *et al.*, 1989; Thomas *et al.*, 2007). Moreover, haematocrit can be affected by both exposure to pesticides (Lopez-Antia*et al.*, 2015a; Singla and Sandhu, 2015) and haematophagous ectoparasites (Whitworth and Bennett, 1992; Simon *et al.*, 2005). We used heparinized capillary tubes to collect ~50 μL of blood from the left brachial vein of 8-day-old nestlings. Approximately 5 μL of blood was smeared for leucocyte counts (see below) and 30 μL was dried on filter paper for DNA sex determination. Molecular sexing was conducted by amplification of chromo-helicase-DNA binding genes and visualization on agarose gel (see Lessard *et al.*, 2014, for more details). The remaining blood was kept in the capillaries on ice and then centrifuged for 7 min at 14 500 g (LWS M24 Hematocrit Centrifuge, LW Scientific, Lawrenceville, GA, USA). Haematocrit was calculated by taking linear measurements in the capillary tube of the packed red cell height and the entire blood column height (in millimetres) with callipers. These measurements were taken twice in a row and the percentage was calculated as the percentage of packed red cell volume to the entire blood column then averaged. The plasma was then pipetted and stored in microtubes at 4°C for up to 8 hours and frozen at −0°C for up to 2 months prior to the BKA assays. Additional blood was sampled in a subsample of the nestlings for other purposes not related to this study, and for those individuals, we measured haematocrit again.

### BKA assays

BKA is a measure of an individual’s blood or plasma’s innate capacity to limit bacterial infection (Matson *et al.*, 2006; Tieleman *et al.*, 2010; Stambaugh *et al.*, 2011). Defence mechanisms linked to this include natural antibodies, complement system and cellular lysis through lysozyme activity. BKA was assessed by looking at nestling plasma defence against *Escherichia coli* using a modified version of Morrison *et al.*’s (2009) protocol. *Escherichia coli* cultures (ATCC 8739) were reconstituted and diluted in tryptic soy broth (TSB) solutions. We mixed 20 mL of this solution with 95 mL of cell culture medium and 5 mL of nestling plasma and then incubated the solution at 40°C for 45 min. We plated 50 μL of the solution on duplicate TSB agars and then incubated plates at 40°C for 24 hours before counting colony forming units (CFUs). We followed the same procedure without nestling plasma as a control. BKA was calculated for each nestling as [(average no. surviving CFUs on plasma-treated plates/average no. CFUs on control plates) × 100]. See Schmitt *et al.* (2017) for more details on assays.

### Leucocyte counts

Leucocyte counts are related to both innate and adaptative mechanisms. Granulocytes and monocytes are part of the innate system and react to any type of pathogen, whereas lymphocytes are linked to adaptative responses to specific pathogens (Coico and Sunshine, 2015). To quantify leucocyte counts, we smeared 5 μL of blood from 8-day-old nestlings on microscope slides, air-dried the samples and then stained them in the laboratory with DipQuick (DipQuick Jovet, CDMV). We used a microscope (Zeiss Axio Observer Z) under a 63× oil-free ocular to differentially count 100 leucocytes (lymphocytes, monocytes and granulocytes). This stain makes it difficult to differentiate between heterophils, eosinophils and basophils; thus, we pooled them as granulocytes (Johnstone *et al.*, 2012). For the leucocyte counts, we randomly selected samples from two nestlings per brood.

### Statistical analyses

Statistical analyses were conducted in the R environment (version 4.0.3). All continuous explanatory variables were standardized to a mean of zero and unit variance. We took a two-step approach where we first used a multivariate analysis to assess the correlations between physiological markers in nestlings (Fig. S1). We then performed univariate analyses separately for each physiological marker where we tested for the effects of pesticides, parasites and their interaction (see below). In some univariate models, we included variables known to potentially affect physiological markers including year, sex (male or female) and the percentage of non-intensive cultures within a 500-m radius around the nest box as a proxy of prey availability (Rioux Paquette *et al.*, 2013; Bellavance *et al.*, 2018). Nestling mass was not included in any models because it was highly correlated with age (*r* = 0.81; variance inflation factor = 3.2) and had no effect on any markers. Nest box was nested within farm identity, so nest box and farm identify were included as random effects to account for the non-independence of observations and hierarchical structure of the data (Bolker *et al.*, 2009).

We used the second-order Akaike information criterion (AICc) to establish and rank a list of candidate models for each marker with the AICcmodavg R package (Mazerolle, 2019). We discuss best models (ΔAICc <2) and report model-averaged predictions and their 95% unconditional confidence intervals while fixing non-focal, numerical explanatory variables at their mean value. The conformity of models to normality and homoscedasticity was assessed visually with the DHARMa R package (Hartig, 2019).

Most pesticides were either not detected (i.e. below limit of detection LOD) or detected at concentrations too low to be estimated (i.e. below limit of quantification LOQ) both at the bolus or farm level (Table S1; Fig. 2A and C), so we could not use concentration of pesticides as an explanatory variable. Instead, for each farm, we used the number of insect boluses contaminated by at least one pesticide as a proxy of pesticide exposure through diet. In our models, we also included the total number of boluses collected on the farm to account for differences in pesticide detection probability among farms. We also considered the number of different pesticides detected at each farm as a proxy for cocktail effects, but this variable was highly correlated with number of contaminated boluses (*r* = 0.91; see Fig. 2B) and led to quantitatively similar results and therefore was not considered further. We estimated parasite load per nestling by summing the number of *Protocalliphora* pupae and puparia found in the nest while controlling for the number of surviving nestlings in the nest in the models.

Nestling age was included only in haematocrit models as other markers were measured at the same age (i.e. 8 ± 1 day). Haematocrit was measured multiple times between Days 7 and 16 in several nestlings and can result in high variability because of a strong link between age and haematocrit. To minimize this, we restricted the age window to 7–12 days. For 166 nestlings, we had two measures of haematocrit per nestling taken at different days. To avoid pseudoreplication, we randomly selected one measure per nestling within this age window. To ensure that this random selection did not create structure in our data, we reran our best fit model 50 times, randomly selecting a new measure per nestling for each iteration (frequency distributions of model estimates and their confidence intervals are in Fig. S2). This allowed us to not include nestling identity as a random effect because we were not investigating within-individual variation for this marker. We fitted linear mixed-effect models with the percentage of red blood cells to total blood volume as the response variable because haematocrit values were weakly correlated with total blood volume (*r* = −0.13; Fig. S3) and total volume was not correlated with any of the explanatory variables (as suggested by Brett, 2004). Only nest box identity was retained for haematocrit in the final model because farm identity explained very little variance (<0.01% of total variance).

For BKA, we used generalized linear models with a binomial distribution and logit link function. We found 57% of the nestlings had negative BKA values so we categorized BKA into 0 and 1 for values ≤0 and >0, respectively (Schmitt *et al.*, 2017). The random effects of nest box and farm identity explained little variance (<0.01% of total variance) in BKA and created singularity problems so they were removed from the final model.

The leucocyte data distribution was overdispersed, so we used generalized linear models with a negative binomial distribution and log link function to separately model each type of leucocyte. The random effects of nest box and farm identity explained little variance (<0.01% of total variance) in each leucocyte model and created singularity problems so they were removed from the final model.

## Results

A summary of our statistical approaches and sample size for each marker is in Table S2. Sample size differed among physiological markers because we did not have enough blood to make all measurements for some nestlings. We assessed haematocrit for 513 nestlings (2013: 128; 2014: 210; 2015: 175), BKA for 507 nestlings (2013: 116; 2014: 201; 2015: 190) and leucocyte counts for 226 nestlings (2013: 46; 2014: 105; 2015: 75), all of which were distributed among a total of 133 broods. We found that 56% of nests were infested by *Protocalliphora* (2013: 57%; 2014: 55%; 2015: 57%) and the mean number (± SD) of parasites (pupae and puparia) per nest was 8.0 ± 12.6 (median: 2; range: 0–59) (2013: 5.7 ± 7.7; 2014: 9.3 ± 15.0; 2015: 8.5 ± 13.1), for an average of 1.8 ± 2.8 *Protocalliphora* per nestling (2013: 1.5 ± 1.8; 2014: 2.0 ± 3.2; 2015: 1.9 ± 3.9).

### Haematocrit

**Figure 3:**
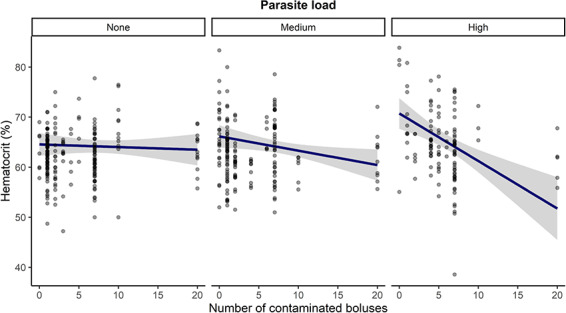
Pesticide and ectoparasite effects on haematocrit in tree swallow nestlings in southern Québec, Canada, 2013–2015. Model-averaged predictions (line) and their 95% CIs (grey area) are based on all candidate models for the year 2015 (Table S3). Although analyses were performed with number of *Protocalliphora* as a numeric variable, nest parasite load is here shown as three categories: None = 0 parasites, Medium = 1–14 parasites and High ≥ 14 parasites. Points represent raw data.

Haematocrit ranged between 38.6% and 83.4%. The best model included the interaction between the number of contaminated boluses and the number of *Protocalliphora* (*w* = 0.40; Table S3). Mean haematocrit [63.3 ± 5.9% (± SD)] did not vary with pesticide exposure in the absence of parasites, but if parasites were present, a negative relationship between haematocrit and pesticide exposure strengthened as parasite load increased (Fig. 3). Interestingly, nestlings exposed to large parasite loads tended to show an increased haematocrit at low pesticide exposure (Fig.3). We also found that males averaged haematocrit is 2% higher than females and that older nestlings had a lower haematocrit than younger nestlings, even within a reduced age window of 7–12 days (Table 1). The second-best model (*w* = 0.34; Table S4) was the full model, which was the best model with year included (see Table S3 for model selection details and S5 for third-best model information).

**Table 1:** Estimates of the best model predicting haematocrit in tree swallow nestlings in southern Québec, Canada, 2013–2015

Variables	Estimate	SE	CI inf	CI sup
**Intercept**	**62.91**	**0.47**	**62.00**	**63.82**
**Number of contaminated boluses**	**−1.32**	**0.43**	**−2.16**	**−0.48**
**Number of *Protocalliphora***	**0.98**	**0.31**	**0.38**	**1.58**
**Sex (male)**	**1.58**	**0.46**	**0.68**	**2.48**
**Total number of boluses**	**1.13**	**0.37**	**0.41**	**1.85**
Brood size	0.38	0.31	−0.24	0.98
% Non-intensive cultures	−0.40	0.46	−1.29	0.49
**Age**	**−1.08**	**0.27**	**−1.60**	**−0.56**
Number of contaminated boluses * number of *Protocalliphora*	**−2.03**	**0.50**	**−2.99**	**−1.06**

See Table S3 for the list of candidate models and their respective weights. Linear mixed model included nest box identity as random effect. Numeric explanatory variables were standardized (zero mean, unit variance). Female was the reference level for ‘Sex’. Estimates for which the 95% CI excludes zero are in bold. ‘*’ refers to interaction between variables

### Bacteria-killing ability

**Figure 4:**
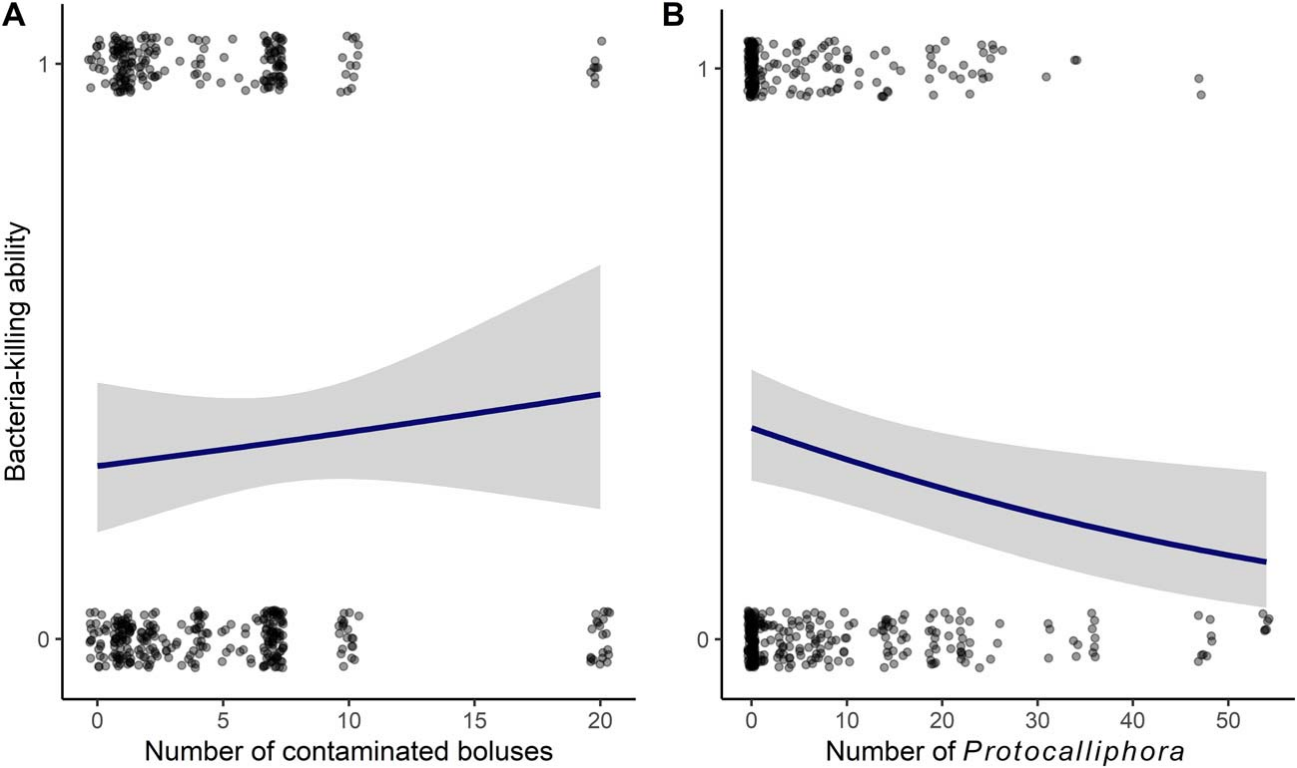
Effect of (A) pesticide exposure (number of contaminated boluses) and (B) ectoparasite load (number of *Protocalliphora*) on BKA of tree swallow nestlings in southern Québec, Canada, 2013–2015. Model-averaged predictions (line) and their 95% CIs (grey area) are based on all candidate models for the year 2015 (Table S4). Points represent raw data.

The best model included number of contaminated boluses, number of *Protocalliphora*, sex and year in addition to control variables (*w* = 0.27; Table S6), but the number of contaminated boluses had no effect on BKA (Table 2). This model indicated that BKA decreased with increasing parasite load (Fig. 4). The second-best model included the interaction between number of contaminated boluses and number of *Protocalliphora*, as well as year, percentage of non-intensive cultures and control variables (*w* = 0.27; Table S7), but only number of *Protocalliphora* and year were related to BKA (see also Table S8 for third-best model). The interaction between pesticide exposure and *Protocalliphora* number was included in the second-best model, but we find no evidence that pesticide exposure modulated the relationship between parasite load and BKA.

**Table 2:** Estimates of the best model predicting the BKA of tree swallow nestlings in southern Québec, Canada, 2013–2015

Variables	Estimate	SE	CI inf	CI sup
**Intercept**	**1.05**	**0.27**	**0.54**	**1.59**
Number of contaminated boluses	0.19	0.17	−0.14	0.53
**Number of *Protocalliphora***	**−0.27**	**0.11**	**−0.50**	**−0.05**
Sex (male)	0.04	0.19	−0.34	0.42
**Year 2014**	**−1.95**	**0.30**	**−2.55**	**−1.37**
**Year 2015**	**−1.85**	**0.34**	**−2.53**	**−1.19**
Brood size	0.12	0.10	−0.08	0.32
**Total number of boluses**	**−0.42**	**0.15**	**−0.72**	**−0.12**
% Non-intensive cultures	0.17	0.14	−0.10	0.43

See Table S4 for the list of candidate models and their respective weights. Generalized linear model was fitted with a binomial distribution and a logit link function. Numeric explanatory variables were standardized (zero mean, unit variance). The reference level for ‘Year’ was 2013 and female for ‘Sex’. Estimates for which the 95% CI excludes zero are in bold.

### Leucocyte counts

Counts ranged from 33 to 94 (mean ± SD = 64.7 ± 13.9) for granulocytes, from 11 to 61 (26.6 ± 13.8) for lymphocytes and from 0 to 47 (9.1 ± 7.7) for monocytes. Several models received equivalent empirical support for both granulocytes and monocytes (see Tables S9 and S10 for model selection details). For granulocytes, the best model (*w* = 0.30; Table 3) included year, sex and percentage of non-intensive cultures. We found a lower granulocyte count for 2014 compared with 2013 and a marginal, negative effect of non-intensive cultures (see Tables S11 and S12 for second- and third-best models). For monocytes, the best model (*w* = 0.61; Table 4) included number of *Protocalliphora* and year, with a lower monocyte count for 2015 compared with 2013. Brood size, used as a control variable for assessing the effect of parasite load in this model, was negatively related to monocyte counts (Table 4; see Table S13 for second-best model). For lymphocytes, the best model (*w* = 0.45; Table 5) included year, sex and non-intensive cultures, with lower lymphocyte counts in 2013 compared with 2014 and 2015 (see Table S14 for model selection details and Table S15 for second-best model).

## Discussion

Our goal was to investigate how exposure to both pesticides and haematophagous *Protocalliphora* ectoparasites would affect tree swallow nestling physiology. We used markers linked to haematology and immunology (innate and acquired) to assess stressor effects in this declining species. Pesticide exposure had a negative effect on haematocrit that grew stronger with increased parasite load, suggesting a detrimental combined effect of the two stressors on nestling aerobic capacity. However, BKA was negatively correlated to only parasite load and none of the leucocyte counts were significantly correlated to pesticide levels or parasite load. We expected the combined exposure to pesticides and parasites to negatively influence all our physiological markers, yet our results suggest that such negative effects could be marker-dependent. Furthermore, low correlations among and between physiological markers and environmental variables revealed in a multivariate analysis suggest context-dependent effects.

**Table 3:** Estimates of the best model predicting the number of granulocytes of tree swallow nestlings in southern Québec, Canada, 2013–2015

Variables	Estimate	SE	CI inf	CI sup
**Intercept**	**4.28**	**0.03**	**4.22**	**4.35**
**Year 2014**	**−0.18**	**0.04**	**−0.25**	**−0.11**
Year 2015	−0.06	0.04	−0.13	0.02
Sex (male)	−0.03	0.03	−0.08	0.02
% Non-intensive cultures	−0.02	0.01	−0.04	0.01

See Table S5 for the list of candidate models and their respective weights. Generalized linear model was fitted with a negative binomial distribution and a log link function. Numeric explanatory variables were standardized (zero mean, unit variance). The reference level for ‘Year’ was 2013 and female for ‘Sex’. Estimates for which the 95% CI excludes zero are in bold.

**Table 4:** Estimates of the best model predicting the number of monocytes of tree swallow nestlings in southern Québec, Canada, 2013–2015

Variables	Estimate	SE	CI inf	CI sup
**Intercept**	**2.25**	**0.09**	**2.07**	**2.44**
Number of *Protocalliphora*	0.03	0.04	−0.05	0.12
Year 2014	0.23	0.11	0.00	0.45
**Year 2015**	**−0.68**	**0.13**	**−0.93**	**−0.43**
**Brood size**	**−0.13**	**0.05**	**−0.21**	**−0.04**
				

See Table S6 for the list of candidate models and their respective weights. Generalized linear model was fitted with a negative binomial distribution and a log link function. Numeric explanatory variables were standardized (zero mean, unit variance). The reference level for ‘Year’ was 2013. Estimates for which the 95% CI excludes zero are in bold.

**Table 5:** Estimates of the best model predicting the number of lymphocytes of tree swallow nestlings in southern Québec, Canada, 2013–2015

Variables	Estimate	SE	CI inf	CI sup
**Intercept**	**2.95**	**0.08**	**2.78**	**3.11**
**Year 2014**	**0.38**	**0.09**	**0.20**	**0.56**
**Year 2015**	**0.36**	**0.10**	**0.17**	**0.54**
Sex (male)	0.06	0.07	−0.08	0.19
% Non-intensive cultures	0.05	0.03	−0.02	0.12

See Table S7 for the list of candidate models and their respective weights. Generalized linear model was fitted with a negative binomial distribution and a log link function. Numeric explanatory variables were standardized (zero mean, unit variance). The reference level for ‘Year’ was 2013 and female for ‘Sex’. Estimates for which the 95% CI excludes zero are in bold.

To our knowledge, no previous study has looked at both pesticide and parasite effects on haematocrit in birds. As expected, the negative influence of pesticides on haematocrit was strongest at high levels of pesticide exposure in highly parasitized nestlings, suggesting a synergistic negative effect of these two stressors. The consumption and accumulation of toxic substances such as pesticides can lead to damaged organs, altered physiological functions and a higher resource cost to maintain homeostasis. For example, some pesticide exposure affects the bone marrow and thereby impacts erythropoiesis (e.g. thiacloprid; Singla and Sandhu, 2015), which can then lead to anaemia (Bishop *et al.*, 1998). Thus, heavily parasitized nestlings can suffer from direct effects such as nutrient and blood loss and further perturbations such as pesticides can then affect their ability to restore red blood cells. Furthermore, we found increased haematocrit values at high parasite loads for nestlings unexposed or slightly exposed to pesticides. Some researchers have argued that birds typically produce red blood cells faster in response to blood loss (Schindler *et al.*, 1987), especially when in good condition and when resources are abundant (Morrison *et al.*, 2009). In fact, O’Brien *et al.* (2001) found that haemoglobin, but not haematocrit, was negatively affected by haematophagous parasitism, underlining active erythropoiesis as a response to blood loss. Nestlings thus seem to cope better with haematophagous parasitism at negligible pesticide exposure, and this response cannot be maintained when pesticide exposure increases in our system creating multiple stressors.

Interestingly, we found that older nestlings had lower haematocrit contrasting with previous studies that generally reported older nestlings had higher haematocrit (Puerta *et al.*, 1989; Potti *et al.*, 1999). Furthermore, our average (± SE) haematocrit values (63.4 ± 0.6%), are higher than other altricial nestlings (38.9–43.2% in *Cyanistes caeruleus*, Simon *et al.*, 2004; 42.3–43.9% in *Sialia sialis*, Carleton, 2008; a median of 39.0% in a different tree swallow populations, Morrison *et al.*, 2009). Jones (2015) also reported that haematocrit ranges between 35% and 55% in birds. To further investigate this unexpected pattern, we looked at the haematocrit of 233 adults in our system for the same time period. Haematocrit for these birds averaged (± SE) 50.2 ± 0.3%, which is very similar to values reported for other passerines (47.6–48.1% in *Ficedula hypoleuca*, Potti, 2007; 50.0% in *S. sialis*, Hannam 2006; 53.3% in *Parus major*, Norte *et al.*, 2009). We measured haematocrit for both adults and nestlings using the same protocol, and this protocol is standard for the previously mentioned field studies. Thus, we are confident in our reported nestling haematocrit values. Dehydration could explain why our reported nestling haematocrits are higher than in previous studies. In our system, nest box openings face southeast and this can lead to warmer temperatures earlier in the day (Ardia *et al.*, 2006). Previous research found dehydration leads to reduced plasma volume resulting in high haematocrit in nestlings (Vleck and Priedkalns, 1985; Campbell, 1995; Ardia, 2013) and might affect blood circulation because of increased viscosity (Norte *et al.*, 2009). We note that haematocrit may be weakly correlated to body condition in birds (Dawson and Bortolotti, 1997a,b; Cuervo *et al.*, 2007), and therefore, our finding should not be generalized to overall individual health. Although tree swallow nestlings in our system do not appear to be anaemic, the synergistic negative effect we found with both pesticide and haematophagous parasite exposure warrants further investigation because it could lead to impaired aerobic capacity, a key component in the foraging ability of these aerial insectivores with further impacts on migration and recruitment (Thomas *et al.*, 2007; Evans *et al.*, 2019).

We also investigated pesticide and haematophagous parasite effects on innate (BKA, granulocytes and monocytes) and adaptive (lymphocytes) immune markers. Immunocompetence is especially important in wildlife because they are constantly exposed to various pathogens. As predicted, we found a negative relationship between parasite load and BKA against *E. coli* though this was not exacerbated by pesticide exposure. Indeed, Eisner Pryor and Casto (2015), one of the few studies that looked at ectoparasite effects on nestling BKA, found that European starling nestlings (*Sturnus vulgaris*) with high haematophagous mites load had lower BKA but only in older broods (15-day-old). They suggested greater investment into growth prior to fledging resulted in a trade-off between growth and immunity towards the end of nestling development. Although we did not assess BKA at different ages, the negative relationship with *Protocalliphora* we report could also reflect a trade-off between immunity and energy investment in growth (Norris and Evans, 2000). An investment in growth could allow nestlings to fledge earlier, which could limit the negative impacts of parasitism (Saino *et al.*, 1998).

Contrary to our expectations, pesticides did not have any effect on BKA nor exacerbated the effect of parasitism on BKA. We have three potential explanations for this finding. First, it is possible that the stress level experienced by tree swallow nestlings was not severe enough for us to detect an effect of pesticides on BKA, alone or in combination with *Protocalliphora* parasitism. Second, our metric for pesticide exposure might not be sufficient to detect pesticide effects. It is difficult to estimate the (cumulative) toxic impact of the cocktail of pesticides to which nestlings in our study area were exposed (e.g. Mineau, 2005; Etterson *et al.*, 2017). Furthermore, we had low pesticide detection rates, despite a sensitive detection rate (i.e. significantly small limit of detection and limit of quantification; Haroune *et al.*, 2015), limiting our ability to quantify pesticide exposure. Third, the parasite load our nestlings experienced might not be high enough for pesticide exposure to have a cumulative negative effect. In a study on tree swallows in Alberta, Canada, Gentes *et al.* (2007) found that brood infested rates were 100% with an average (± SD) parasite load per nestling ranging from 6.8 ± 3.5 in controlled sites to 15.1 ± 7.9 in sites polluted by oil sand exploitation by-products (e.g. polycyclic aromatic hydrocarbons and naphtenic acids). Here, the authors found evidence of a negative combined effect of parasitism and pollution on nestling body mass. However, our parasite load per nestling averaged (± SE) 1.8 ± 0.2 *Protocalliphora* per nestling, which is more than three times lower than what Gentes *et al.* (2007) found and closer to what a different study on parasites and contaminants in great tits (*P. major*) and European pied flycatchers (*F. hypoleuca*) found (Eeva *et al.*, 1994). They found the average (± SE) parasite load per nestling (2.0 ± 0.3 and 2.4 ± 0.4, respectively) was not high enough to have negative effects on growth or survival of nestlings, even in sites polluted with contaminants such as sulphuric oxides and heavy metals (Eeva *et al.*, 1994). We suggest that *Protocalliphora* loads in our system are low enough that nestlings can maintain normal immune function while efficiently coping with detrimental pesticide effects.

We found no relationship between *Protocalliphora* load and leucocyte counts. Though few studies exist on haematophagous parasite effects on these immune markers, we expected a positive relationship because parasite attachment inflicts wounds on the nestlings (Bennett and Whitworth, 1991), leading to a local inflammatory response that can cause an accumulation of leucocytes in peripheral blood. For example, house martins (*Delichon urbica*) infested with louse flies (Diptera: Hippoboscidae) had higher levels of leucocytes, especially lymphocytes, than non-infested individuals (Saino *et al.*, 1998). However, using leucocyte counts to assess immune function in free-ranging animals is criticized because high counts could be due to either an effective immune system or reflect a current infection (Davis *et al.*, 2008). Therefore, the granulocyte to lymphocyte ratio (G/L) is often employed. This ratio is a measure of physiological stress that usually increases after environmental stress exposure. Glucocorticoids respond to the exposure by increasing circulating granulocytes and decreasing circulating lymphocytes (Davis *et al.*, 2008; Ochs and Dawson, 2008; Johnstone *et al.*, 2012). Previous research has found that pesticides (Shutler and Marcogliese, 2011) and parasites (Bonier *et al.*, 2006; Müller *et al.*, 2011) increased the G/L ratio, but we found that neither of these factors was associated with the G/L ratio in our tree swallow nestlings (see Tables S16–S18 for model selection and best models details).

Our results might also be influenced by the way parasitism was quantified. *Protocalliphora* larvae feed on individual nestlings at night (Bennett and Whitworth, 1991) meaning we could not quantify an individual nestling’s parasite load. Here, we estimated it using the average number of parasites per nestling within a nest that assumes equal numbers of *Protocalliphora* on each nestling. However, several studies have found that haematophagous parasites will aggregate on the least immunocompetent nestling of a brood (Christe *et al.*, 1998; Roulin *et al.*, 2003; Simon *et al.*, 2003). Thus, we might have over- or under-estimated *Protocalliphora* parasitism effects for some individuals. Finally, we note that a certain fraction of nestlings died prior to blood sampling and could not be included in our analyses (45 nestlings out of 793 that hatched in our system between 2013 and 2015). These nestlings that did not survive might have had lower physiological markers. For instance, Christe *et al.* (1998) found that dead house martin nestlings tended to have lower leucocyte and red blood cell counts than their fledged siblings at the same age. Tree swallow nestlings that died earlier might have had more pesticide and ectoparasite exposure than the nestlings we measured, leading to an underestimation of the effects caused by these two factors.

## Concluding remarks

We found limited evidence of pesticide or haematophagous ectoparasite effects on tree swallow nestling immunity. However, we reported evidence of a combined effect of pesticides and ectoparasites on haematocrit. This marker-dependent trend underlines the complexity of studying physiological responses in the field. Our study also highlights the importance of considering multiple factors to accurately assess anthropogenic contaminant effects on wildlife. Over the past decades, several aerial insectivore populations showed severe declines in North America (NABCI, 2019; Rosenberg *et al.*, 2019). Pesticides are likely to play an important role in these declines and their interaction with parasites should be considered in future ecotoxicological studies. Because the toxic load of pesticides is still increasing in the environment (Dibartolomeis *et al.*, 2019), we can expect larger impacts on wildlife physiology and increased possibilities of combined effects with parasites. More studies under natural settings are needed to understand both lethal and sublethal pesticide effects on wildlife, especially in declining species (Mineau, 2005).

## Funding

This work was supported by the Fonds de Recherche du Québec–Nature et Technologies (2013-PR-167001 to D.G., F.P. and M.B.); the Natural Sciences and Engineering Research Council of Canada (NSERC) Discovery Grants (327312 to D.G., 261398 to M.B. and 355492 and 05405 to F.P.); and the NSERC Canada Research Chairs program (229221 to F.P.).

## Supplementary Material

revised_suppmat_Sigouin_et_al_coab025
