# Supplementary material for: Agricultural pesticides and ectoparasites: potential combined effects on the physiology of a declining aerial insectivore
Source: Conserv Physiol. 2021 Apr 28;9(1):coab025. doi: 10.1093/conphys/coab025 (PMC8084023; doi:10.1093/conphys/coab025)
Supplement: revised_suppmat_Sigouin_et_al_coab025 [file revised_suppmat_sigouin_et_al_coab025.docx]

Supplementary Material for

Agricultural pesticides and ectoparasites: potential combined effects on the physiology of a declining aerial insectivore

by

Audrey Sigouin, Marc Bélisle, Dany Garant and Fanie Pelletier

Multivariate analyses of biomarkers.

Given the closed data structure of the leucocyte counts that summed up to 100%, we ran a compositional principal component analysis (PCA; Filzmoser *et al*., 2009) with the function pcaCoDa within the robCompositions R package (Hron *et al.*, 2010). This function allows the inclusion of both compositional (number of lymphocytes, monocytes, and granulocytes) and non-compositional (BKA and hematocrit) data into a single PCA. To reduce variation in hematocrit values due to nestling age, we restricted the age window for this measurement to 7, 8, and 9 days (i.e., the age of nestlings at BKA and leucocyte sampling ± 1 day), for a total of 121 nestlings considered in this analysis. We also added the number of insect boluses contaminated at the farm scale and number of *Protocalliphora* in the nest as non-compositional data to assess potential correlations with physiological markers. Because BKA values were not normally distributed, we used the following transformation prior to analysis: -√(1- BKA) (Legendre and Legendre, 1998; Pigeon *et al.*, 2013). Non-compositional data was standardized (zero mean, unit variance), and pcaCoDa function automatically applies an isometric log-ratio transformation to compositional data (Hron *et al.*, 2010).


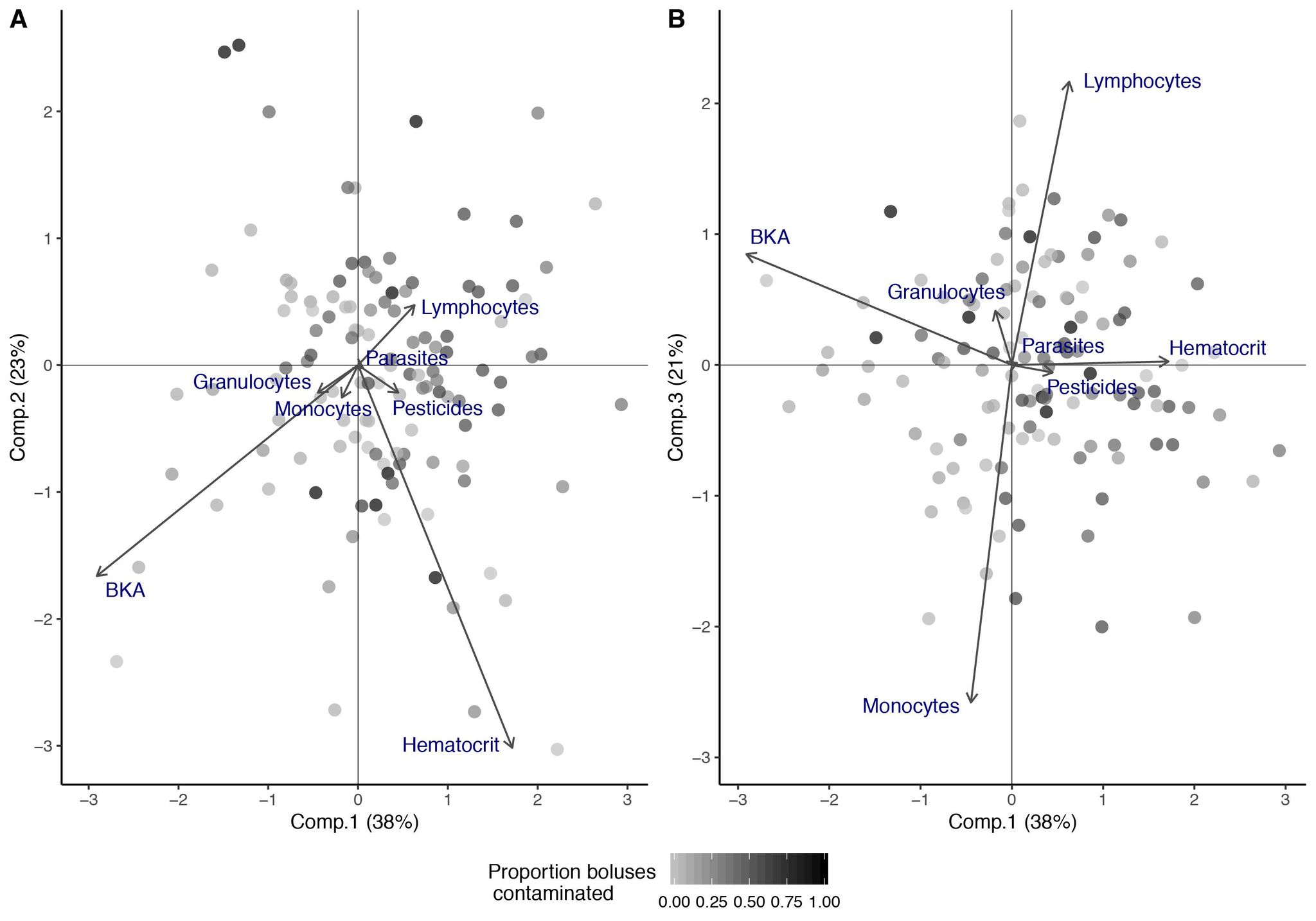
The PCA biplot revealed no clear cluster of points that would have suggested that nestlings showed specific physiological profiles according to their relative exposure to pesticides, as measured by the proportion of boluses contaminated at the farm scale (Figure S1). Most of the variance (82%) was explained by the first three components. Nestling physiology varied mostly according to hematocrit and BKA as these variables had relatively high loadings on PC1 (hematocrit: 0.50; BKA: -0.83) and PC2 (hematocrit: -0.86; BKA: -0.48). Nestlings also varied based on their opposite counts of lymphocytes (0.62) and monocytes (-0.74), which had high loadings on PC3.

**Figure S1: Compositional PCA of physiological markers (hematocrit, BKA, number of granulocytes, number of lymphocytes and number of monocytes) of tree swallow nestlings and environmental data (number of contaminated boluses “Pesticides”, and number of *Protocalliphora* “Parasites”).**

Panel A shows components 1 and 2, which respectively explain 38% and 23% of variance. Panel B shows components 1 and 3, the latest explaining 21% of variance. Points represent nestlings. Color gradient refers to the proportion of boluses contaminated by pesticides at the farm scale (number of contaminated boluses / total number of boluses collected).


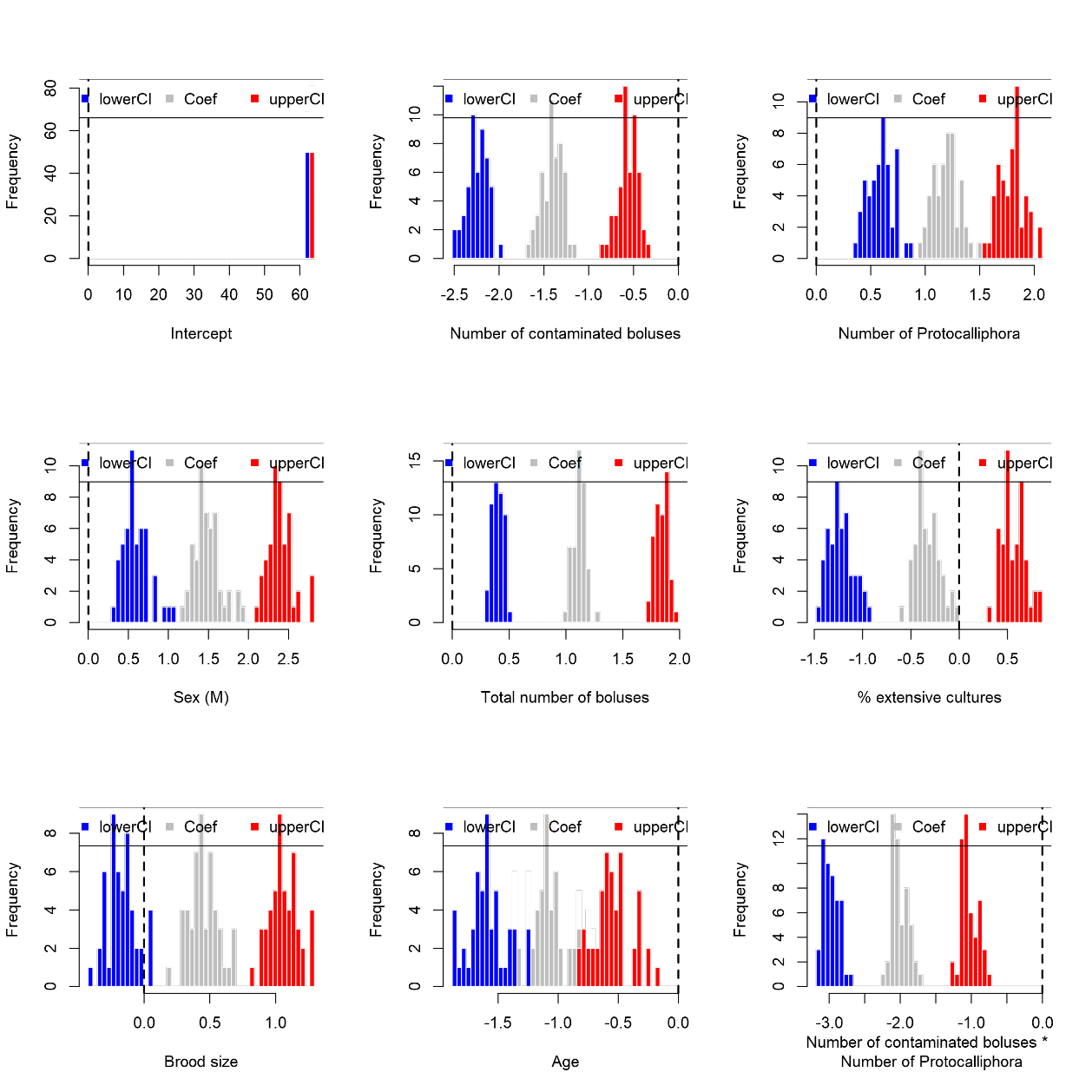
**F****igure S2: Frequency of coefficients (grey bars), lower (blue bars) and upper (red bars) 95 % confidence intervals (CI) obtained with a 50 iterations rerunning of the best model to predict hematocrit measured in tree swallow nestlings in southern Québec, Canada, 2013-2015.**

For each iteration, a single measure of hematocrit was randomly selected for each nestling. Dashed line represents 0. Since CI do not include 0 for parameters of interest (number of contaminated boluses, number of *Protocalliphora* and their interaction), random selection of a single hematocrit measure per nestling does not bias our model interpretation.


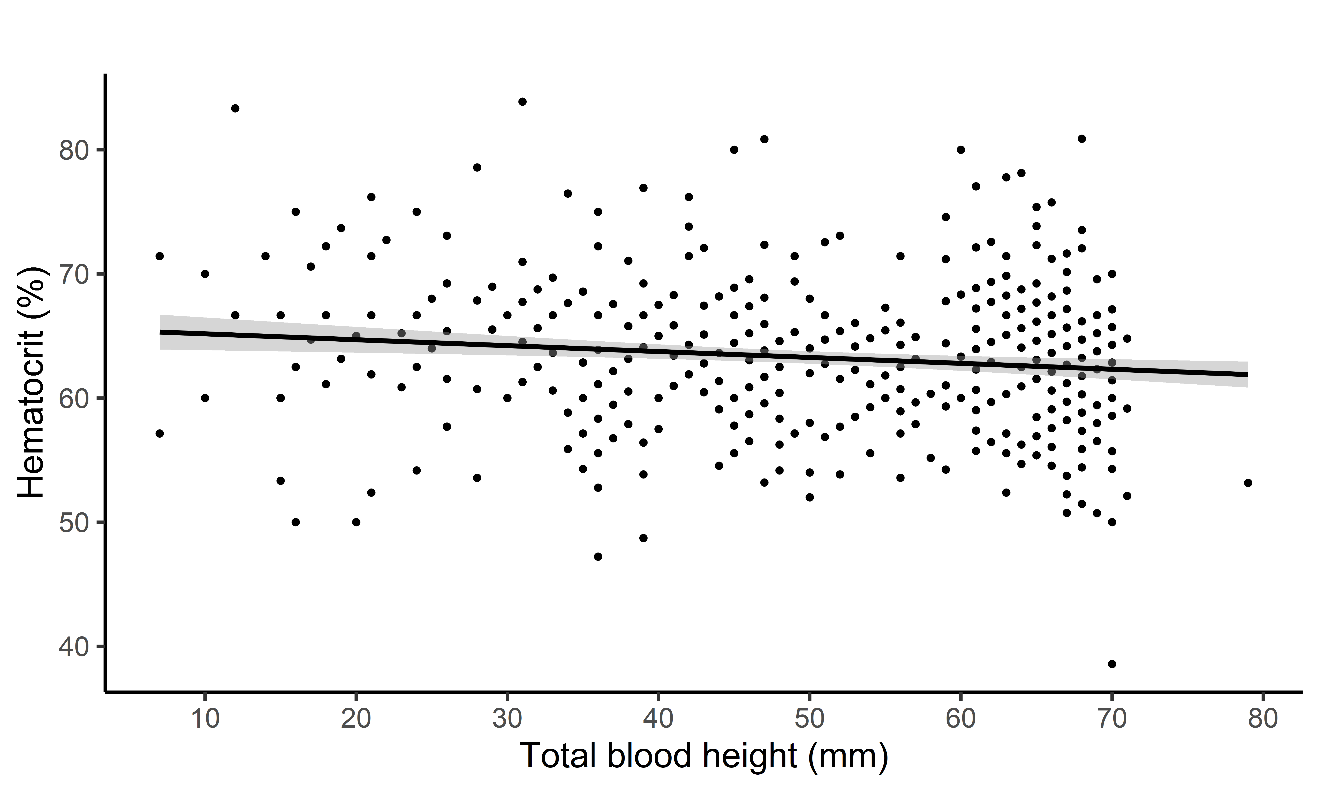


**Figure S3: Hematocrit measured in tree swallow nestlings in 2013-2015 as a function of total blood collected in a capillary tube.**

Correlation is r = -0.13 (p= 0.003). The grey area represents 95 % CIs.

**Table S1 : Limits of detection (LOD) and quantification (LOQ) and detection frequency for 54 pesticide compounds assessed between 2013 and 2015 in insect boluses collected in 10 farms in southern Québec, Canada. Detection frequency refers to the number of detections over the course of the study.**

| Pesticide | LOD  (ng/g) | LOQ  (ng/g) | Frequency | Pesticide | LOD  (ng/g) | LOQ  (ng/g) | Frequency |
| --- | --- | --- | --- | --- | --- | --- | --- |
| One.naphthol | 0.99 | 2.40 | 0 | Thiacloprid | 0.88 | 1.92 | 4 |
| Aldicarb | 1.48 | 5.22 | 5 | Hexazinone | 0.39 | 2.45 | 0 |
| Carbendazim | 0.95 | 2.40 | 1 | Imidacloprid | 2.08 | 5.29 | 4 |
| Pyrimethanil | 2.43 | 5.49 | 0 | Metobromuron | 2.98 | 8.54 | 1 |
| Thiabendazole | 0.78 | 2.10 | 4 | Nitenpyram | 1.08 | 2.77 | 0 |
| Simazine | 1.30 | 3.32 | 1 | Pendimethalin | 0.93 | 2.78 | 3 |
| Carbaryl | 0.87 | 2.54 | 0 | Metolachlor | 1.04 | 2.32 | 45 |
| Dinotefuran | 1.93 | 4.16 | 0 | Imazethapyr | 0.81 | 2.25 | 29 |
| Aldicarb.sulfoxide | 0.76 | 3.31 | 1 | Thiamethoxam | 1.33 | 3.58 | 9 |
| Isoproturon | 1.39 | 3.34 | 0 | Parathion | 4.38 | 14.41 | 10 |
| Chlorotoluron | 1.34 | 3.23 | 0 | Diazinon | 0.99 | 2.26 | 1 |
| O.methoate | 2.64 | 8.02 | 0 | Krezoxim.methyl | 3.34 | 8.84 | 0 |
| Monolinuron | 0.96 | 3.53 | 0 | Azinphos.methyl | 2.44 | 7.06 | 2 |
| Atrazine | 1.61 | 4.44 | 60 | Phosmet | 1.41 | 3.48 | 1 |
| Methabenzthiazuron | 1.35 | 2.97 | 0 | Iprodione | 7.53 | 24.50 | 0 |
| Carbofuran | 0.96 | 2.36 | 1 | Malathion | 4.79 | 9.68 | 0 |
| Acetamiprid | 1.40 | 3.40 | 0 | Boscalid | 1.72 | 5.36 | 0 |
| Aldicarb.sulfone | 2.89 | 7.40 | 0 | Chlorpyrifos | 0.49 | 1.82 | 2 |
| Bendiocarb | 1.79 | 3.69 | 0 | Piperonyl.butoxide | 0.78 | 2.10 | 6 |
| Metoxuron | 1.15 | 2.53 | 0 | Chlorfenvinphos | 2.31 | 6.05 | 0 |
| Dimethoate | 0.84 | 2.20 | 0 | Coumaphos | 2.05 | 4.62 | 0 |
| Terbhutylazine | 1.03 | 2.64 | 0 | Pyraclostrobin | 1.24 | 3.35 | 0 |
| Sebuthylazine | 1.01 | 2.18 | 0 | Permethrin | 1.16 | 2.79 | 0 |
| Diuron | 1.75 | 3.54 | 0 | Trifloxystrobin | 1.31 | 2.97 | 3 |
| Cyanazine | 1.38 | 3.74 | 0 | Spinosad.A | 1.04 | 3.11 | 1 |
| Linuron | 5.62 | 18.82 | 0 | Bentazon | 2.29 | 7.39 | 7 |
| Clothianidin | 1.74 | 5.71 | 22 | Fludioxonil | 10.8 | 31.6 | 0 |

**Table S2: Summary of statistical methods used for candidate model selection of the different physiological markers and their respective sample size.**

| Response variable | Model | Distribution | Link function | N |
| --- | --- | --- | --- | --- |
| Hematocrit (%) | Linear with mixed-effect | Gaussian | Identity | 513 |
| BKA | Generalized | Binomial | Logit | 507 |
| Leucocyte counts  (granulocytes,  lymphocytes,  monocytes) | Generalized | Negative binomial | Log | 233 |

**Table S3 : Candidate models for hematocrit and model selection.**

| Candidate models | Df | AICc | ΔAICc | AICc weight | Cum. weight |
| --- | --- | --- | --- | --- | --- |
| Number contaminated boluses * Number *Protocalliphora* + Sex + % non-intensive cultures | 11 | 3190.60 | 0.00 | 0.40 | 0.40 |
| Number contaminated boluses * Number *Protocalliphora* + Year + Sex + % non-intensive cultures | 13 | 3190.93 | 0.33 | 0.34 | 0.74 |
| Number contaminated boluses * Number *Protocalliphora* + Year + Sex | 12 | 3191.52 | 0.92 | 0.25 | 0.99 |
| Number contaminated boluses * Number *Protocalliphora* + % non-intensive cultures | 10 | 3200.34 | 9.74 | 0.00 | 1.00 |
| Number contaminated boluses + Number *Protocalliphora* + Year + % non-intensive cultures | 12 | 3200.84 | 10.24 | 0.00 | 1.00 |
| Number contaminated boluses + Number *Protocalliphora* + Sex + % non-intensive cultures | 10 | 3205.13 | 14.53 | 0.00 | 1.00 |
| Number *Protocalliphora* + Year + Sex | 12 | 3206.49 | 15.89 | 0.00 | 1.00 |
| Number contaminated boluses + Number *Protocalliphora* + Year + Sex + % non-intensive cultures | 9 | 3208.68 | 18.08 | 0.00 | 1.00 |
| Number *Protocalliphora* + Year + Sex + % non-intensive cultures | 10 | 3210.68 | 20.08 | 0.00 | 1.00 |
| Year + Sex + % non-intensive cultures | 6 | 3214.44 | 23.84 | 0.00 | 1.00 |
| Number contaminated boluses + Number *Protocalliphora* + % non-intensive cultures | 9 | 3214.56 | 23.96 | 0.00 | 1.00 |
| Number contaminated boluses | 8 | 3214.84 | 24.24 | 0.00 | 1.00 |
| Number *Protocalliphora* + Year | 8 | 3217.05 | 26.45 | 0.00 | 1.00 |
| Number *Protocalliphora* | 6 | 3217.67 | 27.07 | 0.00 | 1.00 |
| Number *Protocalliphora* + % non-intensive cultures | 7 | 3219.63 | 29.03 | 0.00 | 1.00 |
| Null | 3 | 3233.47 | 42.87 | 0.00 | 1.00 |

Degree of freedom (df), Akaike information criterion (AICc), the difference of Akaike information criterion between models (ΔAICc), the weighted-Akaike information criterion (AICc weight) and the cumulative weight for each model candidates (Cum. weight) is shown. Random effect includes nest box identity. Control variables are nestling’s age in all model, number of hatchlings for models including number of *Protocalliphora* and total number of boluses for models including number of contaminated boluses.

**Table S4: Estimates of the second-best model predicting hematocrit measured in tree swallow nestlings in southern Québec, Canada, 2013-2015.**

| Variables | Estimate | SE | CI inf | CI sup |
| --- | --- | --- | --- | --- |
| **Intercept** | **62.29** | **0.72** | **60.88** | **63.68** |
| **Number of contaminated boluses** | **-2.04** | **0.59** | **-3.18** | **-0.91** |
| **Number of *Protocalliphora*** | **0.93** | **0.31** | **0.33** | **1.53** |
| Sex (Male) | 0.22 | 0.74 | -1.21 | 1.65 |
| Year 2014 | 1.55 | 0.93 | -0.24 | 3.38 |
| **Year 2015** | **1.59** | **0.46** | **0.70** | **2.50** |
| **Total number of boluses** | **1.60** | **0.48** | **0.67** | **2.52** |
| Brood size | 0.52 | 0.32 | -0.12 | 1.13 |
| % non-intensive cultures | -0.81 | 0.51 | -1.79 | 0.16 |
| **Age** | **-0.93** | **0.28** | **-1.46** | **-0.37** |
| **Number of contaminated boluses * Number of *Protocalliphora*** | **-2.10** | **0.50** | **-3.07** | **-1.13** |

Mixed-effect model (with effect of nest box identity) was fitted with a Gaussian distribution. Numeric explanatory variables were standardized (zero mean, unit variance). The reference level for “Year” was 2013” and female for “Sex”. Estimates for which the 95 % confidence interval excludes zero are in bold. * Refers to interaction between variables

**Table S5: Estimates of the third-best model predicting hematocrit measured in tree swallow nestlings in southern Québec, Canada, 2013-2015.**

| Variables | Estimate | SE | CI inf | CI sup |
| --- | --- | --- | --- | --- |
| **Intercept** | **62.64** | **0.69** | **61.28** | **63.98** |
| **Number of contaminated boluses** | **-1.48** | **0.48** | **-2.40** | **-0.56** |
| **Number of *Protocalliphora*** | **1.03** | **0.31** | **0.44** | **1.62** |
| Year 2014 | -0.05 | 0.72 | -1.45 | 1.35 |
| Year 2015 | 0.86 | 0.83 | -0.74 | 2.50 |
| **Sex (Male)** | **1.58** | **0.46** | **0.68** | **2.48** |
| **Total number of boluses** | **1.19** | **0.41** | **0.40** | **1.98** |
| Brood size | 0.38 | 0.31 | -0.23 | 0.98 |
| **Age** | **-1.04** | **0.27** | **-1.56** | **-0.50** |
| **Number of contaminated boluses * Number of *Protocalliphora*** | **-2.01** | **0.50** | **-2.98** | **-1.04** |

Mixed-effect model (with effect of nest box identity) was fitted with a Gaussian distribution. Numeric explanatory variables were standardized (zero mean, unit variance). The reference level for “Year” was 2013” and female for “Sex”. Estimates for which the 95 % confidence interval excludes zero are in bold.

**Table S6: Candidate models for bacteria-killing ability and model selection.**

| Candidate models | Df | AICc | ΔAICc | AICc weight | Cum. weight |
| --- | --- | --- | --- | --- | --- |
| Number contaminated boluses + Number *Protocalliphora* + Year + Sex | 9 | 639.10 | 0.00 | 0.27 | 0.27 |
| Number contaminated boluses * Number *Protocalliphora* + Year + % non-intensive cultures | 9 | 639.14 | 0.04 | 0.27 | 0.54 |
| Number *Protocalliphora* + Year | 5 | 640.19 | 1.09 | 0.16 | 0.69 |
| Number contaminated boluses * Number *Protocalliphora* + Year + Sex | 9 | 640.60 | 1.50 | 0.13 | 0.82 |
| Number contaminated boluses * Number *Protocalliphora* + Year + Sex + % non-intensive cultures | 10 | 641.18 | 2.08 | 0.10 | 0.92 |
| Number *Protocalliphora* + Year + Sex | 6 | 642.11 | 3.01 | 0.06 | 0.98 |
| Number *Protocalliphora* + Year + Sex + % non-intensive cultures | 7 | 644.10 | 5.00 | 0.02 | 1.00 |
| Year + Sex + % non-intensive cultures | 5 | 649.62 | 10.51 | 0.00 | 1.00 |
| Number *Protocalliphora* | 3 | 680.72 | 41.62 | 0.00 | 1.00 |
| Number *Protocalliphora* + % non-intensive cultures | 4 | 682.34 | 43.23 | 0.00 | 1.00 |
| Number contaminated boluses + Number *Protocalliphora* + % non-intensive cultures | 6 | 683.11 | 44.01 | 0.00 | 1.00 |
| Number contaminated boluses * Number *Protocalliphora* + % non-intensive cultures | 7 | 684.53 | 45.43 | 0.00 | 1.00 |
| Number contaminated boluses + Number *Protocalliphora* + Sex + % non-intensive cultures | 7 | 684.87 | 45.77 | 0.00 | 1.00 |
| Number contaminated boluses * Number *Protocalliphora* + Sex + % non-intensive cultures | 8 | 686.30 | 47.20 | 0.00 | 1.00 |
| Null | 1 | 688.44 | 49.34 | 0.00 | 1.00 |
| Number contaminated boluses | 3 | 688.44 | 49.34 | 0.00 | 1.00 |

Degree of freedom (df), Akaike information criterion (AICc), the difference of Akaike information criterion between models (ΔAICc), the weighted-Akaike information criterion (AICc weight) and the cumulative weight for each model candidates (Cum. weight) is shown. Control variables are number of hatchlings for models including number of *Protocalliphora* and total number of boluses for models including number of contaminated boluses.

**Table S7: Estimates of the second-best model predicting bacteria-killing ability measured in tree swallow nestlings in southern Québec, Canada, 2013-2015.**

| Variables | Estimate | SE | CI inf | CI sup |
| --- | --- | --- | --- | --- |
| **Intercept** | **1.07** | **0.25** | **0.60** | **1.57** |
| Number of contaminated boluses | 0.20 | 0.18 | -0.15 | 0.54 |
| **Number of *Protocalliphora*** | **-0.27** | **0.11** | **-0.50** | **-0.05** |
| **Year 2014** | **-1.95** | **0.30** | **-2.55** | **-1.37** |
| **Year 2015** | **-1.85** | **0.34** | **-2.54** | **-1.19** |
| Brood size | 0.12 | 0.10 | -0.07 | 0.32 |
| **Total number boluses** | **-0.42** | **0.15** | **-0.73** | **-0.12** |
| % non-intensive cultures | 0.17 | 0.14 | -0.10 | 0.43 |
| Number of contaminated boluses * Number of *Protocalliphora* | 0.00 | 0.16 | -0.32 | 0.32 |

Generalized linear model was fitted with a binomial distribution and a logit link function. Numeric explanatory variables were standardized (zero mean, unit variance). The reference level for “Year” was 2013. Estimates for which the 95 % confidence interval excludes zero are in bold.

**Table S8: Estimates of the third-best model predicting bacteria-killing ability measured in tree swallow nestlings in southern Québec, Canada, 2013-2015.**

| Variables | Estimate | SE | CI inf | CI sup |
| --- | --- | --- | --- | --- |
| **Intercept** | **0.75** | **0.20** | **0.36** | **1.16** |
| **Number of *Protocalliphora*** | **-0.33** | **0.11** | **-0.56** | **-0.12** |
| **Year 2014** | **-1.58** | **0.26** | **-2.09** | **-1.08** |
| **Year 2015** | **-1.38** | **0.25** | **-1.89** | **-0.89** |
| Brood size | 0.06 | 0.10 | -0.13 | 0.25 |

Generalized linear model was fitted with a binomial distribution and a logit link function. Numeric explanatory variables were standardized (zero mean, unit variance). The reference level for “Year” was 2013. Estimates for which the 95 % confidence interval excludes zero are in bold.

**Table S9: Candidate models for granulocytes and model selection.**

| Candidate models | Df | AICc | ΔAICc | AICc weight | Cum. weight |
| --- | --- | --- | --- | --- | --- |
| Year + Sex + % non-intensive culture | 6 | 1875.69 | 0.00 | 0.30 | 0.30 |
| Number of *Protocalliphora* + Year | 6 | 1876.23 | 0.54 | 0.23 | 0.53 |
| Number of *Protocalliphora* + Sex + Year | 7 | 1877.14 | 1.44 | 0.15 | 0.67 |
| Number of *Protocalliphora* + % non-intensive cultures | 7 | 1877.14 | 1.44 | 0.15 | 0.82 |
| Number of *Protocalliphora* + Sex + Year + % non-intensive cultures | 8 | 1877.60 | 1.91 | 0.12 | 0.93 |
| Number of contaminated boluses * Number of *Protocalliphora* + Year + Sex | 10 | 1880.65 | 4.96 | 0.03 | 0.96 |
| Number of contaminated boluses + Number of *Protocalliphora* + Year + Sex | 10 | 1881.04 | 5.35 | 0.02 | 0.98 |
| Number of contaminated boluses * Number of *Protocalliphora* + Year + % non-intensive cultures | 10 | 1882.30 | 6.61 | 0.01 | 0.99 |
| Number of contaminated boluses * Number of *Protocalliphora* + Year + Sex + % non-intensive cultures | 11 | 1882.73 | 7.04 | 0.01 | 1.00 |
| Number of contaminated boluses | 4 | 1897.83 | 22.14 | 0.00 | 1.00 |
| Null | 2 | 1897.97 | 22.28 | 0.00 | 1.00 |
| Number of *Protocalliphora* | 4 | 1901.80 | 26.11 | 0.00 | 1.00 |
| Number of contaminated boluses + Number of *Protocalliphora* + % non-intensive cultures | 7 | 1903.46 | 27.77 | 0.00 | 1.00 |
| Number of contaminated boluses * Number of *Protocalliphora* + % non-intensive cultures | 8 | 1905.52 | 29.82 | 0.00 | 1.00 |
| Number of contaminated boluses * Number of *Protocalliphora* + Sex + % non-intensive cultures | 9 | 1906.55 | 30.85 | 0.00 | 1.00 |

Degree of freedom (df), Akaike information criterion (AICc), the difference of Akaike information criterion between models (ΔAICc), the weighted-Akaike information criterion (AICc weight) and the cumulative weight for each model candidates (Cum. weight) is shown. Control variables are number of hatchlings for models including number of *Protocalliphora* and total number of boluses for models including number of contaminated boluses.

**Table S10: Candidate models for monocytes and model selection.**

| Candidate models | Df | AICc | ΔAICc | AICc weight | Cum. weight |
| --- | --- | --- | --- | --- | --- |
| Number of *Protocalliphora* + Year | 6 | 1420.91 | 0.00 | 0.61 | 0.61 |
| Number of *Protocalliphora* + Year + Sex | 7 | 1422.94 | 2.03 | 0.22 | 0.83 |
| Number of *Protocalliphora* + Year + Sex + % non-intensive cultures | 8 | 1425.02 | 4.11 | 0.08 | 0.90 |
| Number of contaminated boluses * Number of *Protocalliphora* + Year + % non-intensive cultures | 10 | 1427.04 | 6.14 | 0.03 | 0.93 |
| Number of contaminated boluses * Number of *Protocalliphora* + Year + Sex | 10 | 1427.13 | 6.22 | 0.03 | 0.96 |
| Year + Sex + % non-intensive cultures | 6 | 1427.82 | 6.92 | 0.02 | 0.98 |
| Number of contaminated boluses + Number of *Protocalliphora* + Year + Sex + % non-intensive cultures | 10 | 1428.60 | 7.69 | 0.01 | 0.99 |
| Number of contaminated boluses * Number of *Protocalliphora* + Year + Sex + % non-intensive cultures | 11 | 1429.24 | 8.33 | 0.01 | 1.00 |
| Null | 2 | 1480.16 | 59.26 | 0.00 | 1.00 |
| Number of contaminated boluses | 4 | 1480.37 | 59.46 | 0.00 | 1.00 |
| Number of contaminated boluses + Number of *Protocalliphora* + % non-intensive cultures | 7 | 1480.72 | 59.81 | 0.00 | 1.00 |
| Number of *Protocalliphora* | 4 | 1481.25 | 60.34 | 0.00 | 1.00 |
| Number of contaminated boluses * Number of *Protocalliphora* + % non-intensive cultures | 8 | 1482.25 | 61.34 | 0.00 | 1.00 |
| Number of *Protocalliphora* + % non-intensive cultures | 5 | 1483.26 | 62.35 | 0.00 | 1.00 |
| Number of contaminated boluses * Number of *Protocalliphora* + Sex + % non-intensive cultures | 9 | 1484.19 | 63.28 | 0.00 | 1.00 |

Degree of freedom (df), Akaike information criterion (AICc), the difference of Akaike information criterion between models (ΔAICc), the weighted-Akaike information criterion (AICc weight) and the cumulative weight for each model candidates (Cum. weight) is shown. Control variables are number of hatchlings for models including number of *Protocalliphora* and total number of boluses for models including number of contaminated boluses.

**Table S11: Estimates of the second-best model predicting granulocyte number measured in tree swallow nestlings in southern Québec, Canada, 2013-2015.**

| Variables | Estimate | SE | CI inf | CI sup |
| --- | --- | --- | --- | --- |
| **Intercept** | **4.27** | **0.03** | **4.22** | **4.33** |
| Number of *Protocalliphora* | 0.01 | 0.01 | -0.02 | 0.04 |
| **Year 2014** | **-0.18** | **0.04** | **-0.26** | **-0.12** |
| Year 2015 | -0.06 | 0.04 | -0.13 | 0.01 |
| Brood size | 0.02 | 0.01 | -0.01 | 0.04 |

Generalized linear model was fitted with a negative binomial distribution and a log link function. Numeric explanatory variables were standardized (zero mean, unit variance). The reference level for “Year” was 2013. Estimates for which the 95 % confidence interval excludes zero are in bold.

**Table S12: Estimates of the third-best model predicting granulocyte number measured in tree swallow nestlings in southern Québec, Canada, 2013-2015.**

| Variables | Estimate | SE | CI inf | CI sup |
| --- | --- | --- | --- | --- |
| **Intercept** | **4.29** | **0.03** | **4.22** | **4.35** |
| Number of *Protocalliphora* | 0.01 | 0.01 | -0.01 | 0.04 |
| Sex (Male) | -0.03 | 0.03 | -0.08 | 0.02 |
| **Year 2014** | **-0.19** | **0.04** | **-0.26** | **-0.12** |
| Year 2015 | -0.06 | 0.04 | -0.13 | 0.01 |
| Brood size | 0.02 | 0.01 | -0.01 | 0.04 |

Generalized linear model was fitted with a negative binomial distribution and a log link function. Numeric explanatory variables were standardized (zero mean, unit variance). The reference level for “Year” was 2013 and female for “Sex”. Estimates for which the 95 % confidence interval excludes zero are in bold.

**Table S13: Estimates of the second-best model predicting monocyte number measured in tree swallow nestlings in southern Québec, Canada, 2013-2015.**

| Variables | Estimate | SE | CI inf | CI sup |
| --- | --- | --- | --- | --- |
| **Intercept** | **2.24** | **0.11** | **2.03** | **2.44** |
| Number of *Protocalliphora* | 0.03 | 0.04 | -0.05 | 0.12 |
| Sex (Male) | 0.03 | 0.09 | -0.15 | 0.20 |
| Year 2014 | 0.23 | 0.11 | 0.00 | 0.45 |
| **Year 2015** | **-0.68** | **0.13** | **-0.93** | **-0.43** |
| **Brood size** | **-0.13** | **0.05** | **-0.21** | **-0.04** |

Generalized linear model was fitted with a negative binomial distribution and a log link function. Numeric explanatory variables were standardized (zero mean, unit variance). The reference level for “Year” was 2013 and female for “Sex”. Estimates for which the 95 % confidence interval excludes zero are in bold.

**Table S14: Candidate models for lymphocytes and model selection.**

| Candidate models | Df | AICc | ΔAICc | AICc weight | Cum. weight |
| --- | --- | --- | --- | --- | --- |
| Year + Sex + % non-intensive cultures | 6 | 1851.76 | 0.00 | 0.45 | 0.45 |
| Number of *Protocalliphora* + Year | 6 | 1853.32 | 1.56 | 0.20 | 0.65 |
| Number of *Protocalliphora* + Sex + Year | 7 | 1854.82 | 3.06 | 0.10 | 0.75 |
| Number of *Protocalliphora* + % non-intensive cultures | 7 | 1854.82 | 3.06 | 0.10 | 0.84 |
| Number of *Protocalliphora* + Sex + Year + % non-intensive cultures | 8 | 1855.28 | 3.51 | 0.08 | 0.92 |
| Number of contaminated boluses * Number of *Protocalliphora* + Year + Sex | 10 | 1857.09 | 5.33 | 0.03 | 0.95 |
| Number of contaminated boluses * Number of *Protocalliphora* + Year + % non-intensive cultures | 10 | 1858.06 | 6.30 | 0.02 | 0.97 |
| Number of contaminated boluses + Number of *Protocalliphora* + Year + Sex + % non-intensive cultures | 10 | 1858.31 | 6.55 | 0.02 | 0.99 |
| Number of contaminated boluses * Number of *Protocalliphora* + Year + Sex + % non-intensive cultures | 11 | 1859.17 | 7.41 | 0.01 | 1.00 |
| Null | 2 | 1863.68 | 11.92 | 0.00 | 1.00 |
| Number of contaminated boluses | 4 | 1865.79 | 14.03 | 0.00 | 1.00 |
| Number of *Protocalliphora* | 4 | 1866.39 | 14.63 | 0.00 | 1.00 |
| Number of contaminated boluses + Number of *Protocalliphora* + % non-intensive cultures | 7 | 1868.90 | 17.14 | 0.00 | 1.00 |
| Number of contaminated boluses * Number of *Protocalliphora* + % non-intensive cultures | 8 | 1870.55 | 18.78 | 0.00 | 1.00 |
| Number of contaminated boluses * Number of *Protocalliphora* + Sex + % non-intensive cultures | 9 | 1872.14 | 20.38 | 0.00 | 1.00 |

Degree of freedom (df), Akaike information criterion (AICc), the difference of Akaike information criterion between models (ΔAICc), the weighted-Akaike information criterion (AICc weight) and the cumulative weight for each model candidates (Cum. weight) is shown. Control variables are number of hatchlings for models including number of *Protocalliphora* and total number of boluses for models including number of contaminated boluses.

**Table S15: Estimates of the second-best model predicting lymphocyte number measured in tree swallow nestlings in southern Québec, Canada, 2013-2015.**

| Variables | Estimate | SE | CI inf | CI sup |
| --- | --- | --- | --- | --- |
| **Intercept** | **2.97** | **0.08** | **2.83** | **3.13** |
| Number of *Protocalliphora* | -0.04 | 0.03 | -0.11 | 0.03 |
| **Year 2014** | **0.39** | **0.09** | **0.20** | **0.57** |
| **Year 2015** | **0.35** | **0.10** | **0.16** | **0.54** |
| Brood Size | 0.00 | 0.03 | -0.07 | 0.07 |

Generalized linear model was fitted with a negative binomial distribution and a log link function. Numeric explanatory variables were standardized (zero mean, unit variance). The reference level for “Year” was 2013. Estimates for which the 95 % confidence interval excludes zero are in bold.

**Table S16: Candidate models for granulocyte/lymphocyte ratio and model selection.**

| Candidate models | Df | AICc | ΔAICc | AICc weight | Cum. weight |
| --- | --- | --- | --- | --- | --- |
| Number of *Protocalliphora* + Year | 7 | 1571.13 | 0.00 | 0.55 | 0.55 |
| Number of *Protocalliphora* + Year + Sex | 8 | 1572.64 | 1.52 | 0.26 | 0.81 |
| Number of *Protocalliphora* + Year + Sex + % non-intensive cultures | 9 | 1574.76 | 3.63 | 0.09 | 0.90 |
| Year + Sex + % non-intensive cultures | 7 | 1575.22 | 4.10 | 0.07 | 0.97 |
| Number of contaminated boluses + Number of *Protocalliphora* + Year + Sex + % non-intensive cultures | 11 | 1579.09 | 7.96 | 0.01 | 0.98 |
| Number of contaminated boluses * Number of *Protocalliphora* + Year + Sex | 11 | 1579.11 | 7.99 | 0.01 | 0.99 |
| Number of contaminated boluses * Number of *Protocalliphora* + Year + % non-intensive cultures | 11 | 1579.61 | 8.48 | 0.01 | 1.00 |
| Number of contaminated boluses * Number of *Protocalliphora* + Year + Sex + % non-intensive cultures | 12 | 1581.26 | 10.14 | 0.00 | 1.00 |
| Number of contaminated boluses | 5 | 1604.57 | 33.45 | 0.00 | 1.00 |
| Number of *Protocalliphora* | 5 | 1606.26 | 35.13 | 0.00 | 1.00 |
| Number of *Protocalliphora* + % non-intensive cultures | 6 | 1608.36 | 37.24 | 0.00 | 1.00 |
| Number of contaminated boluses + Number of *Protocalliphora* + % non-intensive cultures | 8 | 1608.55 | 37.43 | 0.00 | 1.00 |
| Number of contaminated boluses + Number of *Protocalliphora* + Sex + % non-intensive cultures | 9 | 1610.03 | 38.90 | 0.00 | 1.00 |
| Number of contaminated boluses * Number of *Protocalliphora* + % non-intensive cultures | 9 | 1610.60 | 39.48 | 0.00 | 1.00 |
| Number of contaminated boluses * Number of *Protocalliphora* + Sex + % non-intensive cultures | 10 | 1612.11 | 40.99 | 0.00 | 1.00 |
| Null | 2 | 1897.97 | 326.84 | 0.00 | 1.00 |

Degree of freedom (df), Akaike information criterion (AICc), the difference of Akaike information criterion between models (ΔAICc), the weighted-Akaike information criterion (AICc weight) and the cumulative weight for each model candidates (Cum. weight) is shown. Ratio was modelized with number of granulocytes as the response variable and number of lymphocytes as a fixed effect in all models. Control variables are number of hatchlings for models including number of *Protocalliphora* and total number of boluses for models including number of contaminated boluses.

**Table S17: Estimates of the best model predicting granulocyte/ lymphocyte ratio measured in tree swallow nestlings in southern Québec, Canada, 2013-2015.**

| Variables | Estimate | SE | CI inf | CI sup |
| --- | --- | --- | --- | --- |
| **Intercept** | **4.17** | **0.02** | **4.13** | **4.20** |
| Number of Protocallihpora | 0.00 | 0.01 | -0.02 | 0.01 |
| **Year 2014** | **-0.07** | **0.02** | **-0.11** | **-0.02** |
| **Year 2015** | **0.05** | **0.02** | **0.01** | **0.10** |
| Brood size | 0.02 | 0.01 | 0.00 | 0.03 |
| **Number of lymphocytes** | **-0.19** | **0.01** | **-0.21** | **-0.17** |

Generalized linear model was fitted with a negative binomial distribution and a log link function, with number of granulocytes as the response variable and number of lymphocytes as a fixed effect in all models. Numeric explanatory variables were standardized (zero mean, unit variance). The reference level for “Year” was 2013. Estimates for which the confidence 95 % interval excludes zero are in bold.

**Table S18: Estimates of the second-best model predicting granulocyte/ lymphocyte ratio measured in tree swallow nestlings in southern Québec, Canada, 2013-2015.**

| Variables | Estimate | SE | CI inf | CI sup |
| --- | --- | --- | --- | --- |
| **Intercept** | **4.17** | **0.02** | **4.13** | **4.21** |
| Number of *Protocalliphora* | 0.00 | 0.01 | -0.02 | 0.01 |
| Sex (Male) | -0.01 | 0.02 | -0.05 | 0.02 |
| **Year 2014** | **-0.07** | **0.02** | **-0.11** | **-0.02** |
| **Year 2015** | **0.05** | **0.02** | **0.01** | **0.09** |
| Brood size | 0.02 | 0.01 | 0.00 | 0.03 |
| **Number of lymphocytes** | **-0.19** | **0.01** | **-0.21** | **-0.17** |

Generalized linear model was fitted with a negative binomial distribution and a log link function, with number of granulocytes as the response variable and number of lymphocytes as a fixed effect in all models. Numeric explanatory variables were standardized (zero mean, unit variance). The reference level for “Year” was 2013 and female for “Sex. Estimates for which the 95 % confidence interval excludes zero are in bold.

# Supplementary references

Filzmoser P, Hron K, Reimann C (2009) Principal component analysis for compositional data with outliers. Environmetrics 20: 621–632.

Hron K, Templ M, Filzmoser P (2010) Exploratory compositional data analysis using the R-package robCompositions. In: Ninth International Conference Data Analysis and Modeling. pp 179–186.

Legendre P, Legendre L (1998) Numerical Ecology. New-York: Elsevier.

Pigeon G, Bélisle M, Garant D, Cohen AA, Pelletier F (2013) Ecological immunology in a fluctuating environment: An integrative analysis of tree swallow nestling immune defense. Ecol Evol 3: 1091–1103.
